# Synaptic output from suprachiasmatic nucleus cholecystokinin neurons regulates locomotor rhythmicity

**DOI:** 10.3389/fnins.2026.1882096

**Published:** 2026-07-03

**Authors:** Wanli Chen, Zhixiong Ma, Xiaoqing Hao, Shikang Guan, Xin Diao, Yuehong Huang, Ruogu Liu, Zhengran Li, Bingbing Xue, Tongfei A. Wang

**Affiliations:** 1 State Key Laboratory of Cognitive Neuroscience and Learning and IDG/McGovern Institute for Brain Research, Beijing Normal University, Beijing, China; 2 Beijing Institute for Brain Research, Chinese Academy of Medical Sciences and Peking Union Medical College, Beijing, China; 3 Chinese Institute for Brain Research, Beijing (CIBR), Beijing, China; 4 Beijing Key Laboratory of Brain Science and Brain-Machine Interface, Beijing, China; 5 School of Basic Medical Sciences, Capital Medical University, Beijing, China; 6 College of Biological Sciences, China Agricultural University, Beijing, China; 7 Academy for Advanced Interdisciplinary Studies, Peking University, Beijing, China; 8 Brain Cognition and Brain Disease Institute, Shenzhen Institutes of Advanced Technology, Chinese Academy of Sciences, Shenzhen, China

**Keywords:** cholecystokinin, circadian rhythm, locomotor activity, suprachiasmatic nucleus, tetanus toxin

## Abstract

**Background:**

The mammalian suprachiasmatic nucleus (SCN) serves as the master circadian pacemaker, which coordinates daily behavioral and physiological rhythms through functionally diverse neuronal subtypes. Cholecystokinin (CCK) is expressed in a subset of SCN neurons; however, its role in locomotor activity rhythms remains poorly understood.

**Methods:**

To study the functional contribution of SCN CCK-expressing (SCN^CCK^) neurons, we selectively blocked synaptic transmission by injecting a Cre-dependent tetanus toxin (TeNT) viral vector into the SCN of CCK-IRES-Cre mice. Before and after the virus injection, spontaneous locomotor activity was continuously recorded under a 12:12 h light–dark (LD) cycle. Subsequently, we used Cre-dependent fluorescent reporter (mYongHong) to label SCN^CCK^ neurons and performed whole-brain projection mapping to characterize their downstream connectivity.

**Results:**

Synaptic inhibition of SCN^CCK^ neurons significantly attenuated the strength of locomotor rhythmicity, resulting in reduced rhythm organization and a more uniform distribution of activity. This disruption was mainly driven by a significant decrease in dark-phase locomotor activity, while light-phase activity remained unchanged. Anatomically, SCN^CCK^ neurons are widely projected along the anterior and posterior axes to multiple hypothalamic, thalamic, and limbic regions, including the medial preoptic area (MPA), paraventricular thalamic nucleus (PVT), paraventricular hypothalamic nucleus (PVH), anterior hypothalamic area (AHC), dorsomedial hypothalamic nucleus (DMH), ventromedial hypothalamic nucleus (VMH), and medial amygdala nucleus (MeA). Quantitative analysis revealed projections to these downstream regions, with moderate variation in projection density across targets.

**Conclusion:**

Together, these findings identify SCN^CCK^ neurons as an important neuronal subpopulation, which contributes to the robustness and consolidation of spontaneous locomotor rhythms, likely through a wide range of downstream circuits.

## 1 Introduction

The SCN is the central circadian pacemaker of mammals, coordinating daily physiological and behavioral rhythms, including locomotor activity (Reppert and Weaver, 2002; Mohawk et al., 2012; Takahashi, 2017). Individual SCN neurons contain cell-autonomous molecular clocks, and their network interactions generate coherent circadian output, driving overt behavioral rhythms (Welsh et al., 2010; Hastings et al., 2018). The disruption of SCN function leads to profound alterations in daily activity patterns, highlighting its essential role in organizing temporal behavior (Moore and Eichler, 1972; Stephan and Zucker, 1972; Ralph et al., 1990). Locomotor activity is one of the most commonly used physiological indicators for the study of circadian rhythms; however, the neural mechanisms underlying circadian locomotor rhythms remain incompletely understood.

Accumulating evidence indicates that the SCN is not a homogeneous structure, but consists of distinct neuronal subtypes with specialized functions. Vasoactive intestinal polypeptide (VIP) neurons are critical for photic entrainment and synchrony within the SCN network, as loss of VIP signaling disrupts circadian locomotor rhythms (Harmar et al., 2002; Aton et al., 2005; Jones et al., 2018). Arginine vasopressin (AVP) neurons are mainly located in the dorsal SCN, which contribute to the accuracy and stability of circadian rhythms (Guzmán-Ruiz et al., 2015; Gizowski et al., 2016). These findings suggest that different SCN neuronal populations may selectively regulate specific aspects of circadian behavior (Morin, 2013).

CCK is expressed in a subset of SCN neurons, which are neurochemically distinct from VIP and AVP populations (Hannibal et al., 2010; Wen et al., 2020). Although CCK neurons have been widely studied for their roles in feeding, arousal, and motivation in other brain regions, their functions within the SCN remains poorly understood (Rotzinger and Vaccarino, 2003; Warrilow et al., 2023). Recent studies reported that SCN^CCK^ neurons show activity patterns associated with nocturnal locomotor behavior, which raise the possibility that these neurons contribute to the regulation of locomotor activity during the active phase (Xie et al., 2023). Notably, Xie further demonstrated that tetanus toxin (TeNT)-mediated inhibition of SCN^CCK^ neurons alters circadian behavioral adaptation under long photoperiod conditions, suggesting a role of these neurons in regulating seasonal plasticity of locomotor rhythms. However, these observations remain largely correlative, and it is not clear whether synaptic output from SCN^CCK^ neurons is required for animals’ daily locomotor activity rhythmicity.

In the present study, we addressed these questions by selectively inhibiting synaptic transmission from SCN^CCK^ neurons. Then, we evaluated the impact of this manipulation on locomotor activity. In parallel, we mapped the axonal projections of SCN^CCK^ neurons using viral labeling to provide an anatomical framework for potential downstream targets. These experiments were designed to define the functional contribution of SCN^CCK^ neurons to locomotor activity regulation and to characterize their underlying anatomical projections.

## 2 Methods

### 2.1 Animals

All animal care and experimental procedures were conducted in accordance with the guidelines of the Institutional Animal Care and Use Committee of the Chinese Institute for Brain Research (CIBR), Beijing (Animal Protocol: CIBR-IACUC-069). CCK-IRES-Cre mice (JAX #012706) were obtained from The Jackson Laboratory. Wild-type (WT) littermates on the C57BL/6J background were obtained from the Laboratory Animal Resource Center for animal care and husbandry. Both male and female mice aged 8–12 weeks were used in this study. Animals were randomly assigned to experimental groups without sex-based stratification, and sex was not included as a biological variable in downstream analyses. WT mice and CCK-IRES-Cre mice were subjected to the same housing conditions and experimental procedures unless otherwise specified. Animals were maintained under controlled environmental conditions, ambient temperature was maintained at 23–25 °C, and the relative humidity was maintained at 40–50%. Mice were housed in light-controlled cabinets under a 12 h light/12 h dark cycle, with ad libitum access to food and water throughout the study. For standardized circadian rhythm measurements, animals were single-housed to ensure accurate behavioral recordings.

### 2.2 Surgical procedures

In this study, all adeno-associated viruses (AAVs) used were packaged by the Vector Core Facility of CIBR. Mice were anesthetized with isoflurane (5% for induction and 1–1.5% for maintenance), and ophthalmic ointment was applied to prevent corneal drying. Animals were fixed in a stereotaxic frame (RWD Instruments, 71,000), and the surgical field was sterilized with 75% ethanol. The skull was exposed and leveled using a nose bar and bilateral ear bars. A total volume of 30 nL of either AAV2/9-CMV-DIO-TeNT-2A-GFP (21 × 10^12^ GC/mL) or AAV2/9-CAG-DIO-mYongHong (10.2 × 10^12^ GC/mL) was stereotaxically injected into the suprachiasmatic nucleus (SCN; angle 20°, AP -0.4 mm, ML 2.2 mm, DV -5.5 mm). For functional experiments, AAV2/9-CMV-DIO-TeNT-2A-GFP was injected bilaterally into the SCN, *n =* 7 mice per group were used. For axonal tracing experiments, AAV2/9-CAG-DIO-mYongHong was injected unilaterally into the SCN, *n =* 4 mice were used. Injections were performed using a microsyringe pump (Nanoliter 2000 injector, WPI) at a rate of 10 nL/min. The injection needle was maintained in position for 6 min after delivery and subsequently withdrawn at 0.1 mm/s. Penicillin (20,000 U/kg, subcutaneous) and meloxicam (1 mg/kg, subcutaneous) were administered subcutaneously immediately after surgery for infection prevention and postoperative analgesia. Animals were allowed a minimum recovery period of one week to ensure viral expression and full recovery prior to subsequent behavioral experiments. Injection sites were verified histologically. The projection-mapping experiments were conducted in an independent cohort of CCK-IRES-Cre mice that was not used for functional manipulation or behavioral experiments.

### 2.3 Histology

Two sectioning strategies were adopted according to experimental requirements. Mice were deeply anesthetized and transcardially perfused with saline, followed by 4% paraformaldehyde (PFA) in phosphate-buffered saline (PBS, pH ~ 7.4). Brains were dissected and post-fixed in 4% PFA for at least 16 h at 4 °C. For verification of viral injection sites in CCK-IRES-Cre mice injected with AAV-DIO-TeNT (*n =* 7 mice), brains were processed for region-specific analysis, coronal sections (50 μm) were prepared using a vibratome (Leica, VT1200 S) and collected sequentially into 24-well plates containing ice-cold PBS. Sections were subsequently mounted onto glass slides, cover slipped, and sealed with nail polish. For whole-brain projection mapping in CCK-IRES-Cre mice injected with AAV-DIO-mYongHong (*n =* 4 mice), brains were processed for comprehensive anatomical preservation. After fixation, brains were cryoprotected in 30% sucrose in PBS at 4 °C with gentle agitation until fully submerged, embedded in OCT compound, and frozen in a cryostat chamber (Leica, CM3050 S). Coronal cryosections (50 μm) were mounted on adhesion microscope slides, cover slipped and sealed with nail polish. Slides were scanned using a slide scanner (Olympus, VS120) with a 10 × objective using the appropriate fluorescence channels for signal detection. GFP fluorescence was acquired using the 488 nm excitation channel, whereas mYongHong fluorescence was acquired using the 561 nm excitation channel. All images were acquired using identical scanning settings across samples to ensure consistency of signal intensity and downstream quantitative analysis. Signal quantification and analysis were performed using QuPath (v0.5.1).

### 2.4 Axon density calculation

Images acquired using the VS120 slide scanner were imported into QuPath for analysis. All image files were anonymized prior to analysis by removing group-identifying information and assigning randomized codes to each dataset. The experimenter responsible for image processing, Regions of interest (ROI) definition, annotation, and quantification was blinded to experimental group identity during the entire analysis workflow. Contrast adjustments were applied uniformly across all images using identical parameters to ensure clear visualization of axonal structures. High-magnification views were generated, and the irregular annotation tool was used to manually label axon-positive and non-axonal pixels as training data. A pixel classifier was subsequently trained in QuPath using the Train Pixel Classifier module with the Artificial Neural Network (ANN) algorithm. Training was performed at full image resolution with feature extraction based on Gaussian smoothing, local mean, and local variance. Local normalization was applied using an analysis scale of 8 μm, and identical classifier settings were used across all brain regions and animals. ROIs were delineated based on Paxinos and Franklin’s *The Mouse Brain in Stereotaxic Coordinates* (5th edition), using matched coronal levels across animals. Specifically, representative sections were selected at defined anterior–posterior levels corresponding to each target region, including the MPA (Bregma +0.13 mm; atlas Figure 30), PVT (−0.35 mm; atlas Figure 34), PVH (−0.59 mm; atlas Figure 36), AHC (−0.83 mm; atlas Figure 38), VMH (−1.55 mm; atlas Figure 44), DMH (−1.79 mm; atlas Figure 46), and MeA (−1.23 mm; atlas Figure 41) in Paxinos and Franklin’s *The Mouse Brain in Stereotaxic Coordinates* (5th edition). For each region, multiple sections per brain were analyzed using consistent ROI boundaries applied across all animals and experimental groups to ensure anatomical comparability. Axon density was calculated as the ratio of axon-positive area to the total ROI area. For each ROI, axon-positive and background signals were quantified using the trained pixel classifier, and measurements were extracted from all annotations within each region. All images were processed using an identical, predefined QuPath analysis pipeline to ensure reproducibility. Quantification was performed on *n =* 4 mice per group.

### 2.5 Locomotor activity recording

**Figure 1:**
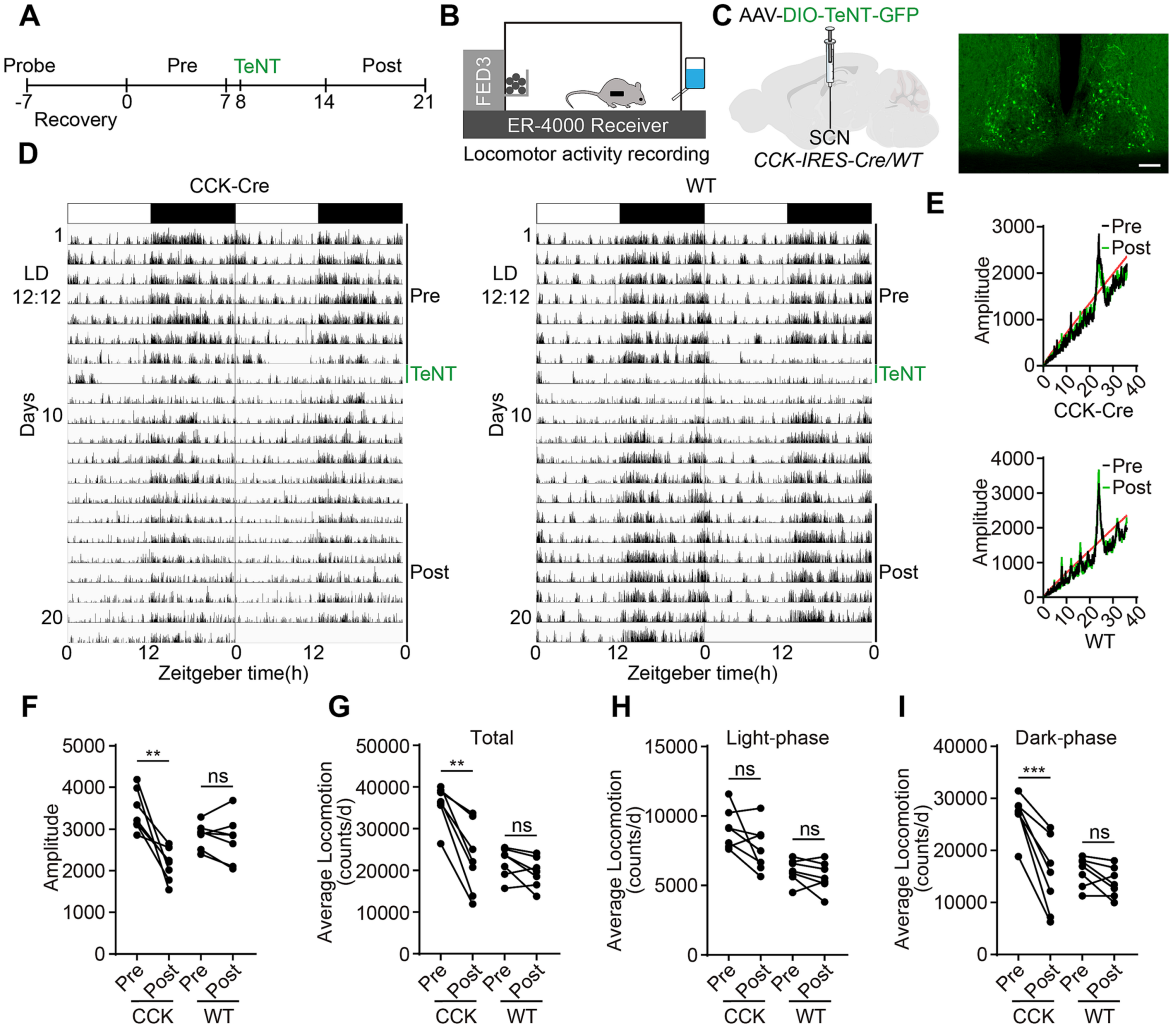
Inhibiting SCN^CCK^ neurons significantly dampens spontaneous locomotor activity rhythms in mice. **(A)** Experimental timeline for locomotor activity recordings be-fore (Pre), during (TeNT expression), and after viral inhibition (Post), “Probe” refers to the implantable telemetry transmitter used for continuous monitoring of locomotor activity. **(B)** Schematic diagram of the experimental setup for monitoring locomotor activity in CCK-IRES-Cre mice. **(C)** Bilateral AAV2/9-CMV-DIO-TeNT-GFP injection into the SCN of CCK-IRES-Cre mice and representative GFP fluorescence confirming localized expression in SCN neurons. Scale bar, 100 μm. **(D)** Representative double-plotted actograms showing locomotor activity in TeNT-treated CCK-Cre mice and WT controls under 12:12 LD conditions. Individual actograms for all animals are provided in the Supplementary Figures S2A, S3A. **(E)** Chi-square periodogram analysis of the same representative CCK-TeNT mouse shown in **(D)**, together with the corresponding periodogram from a WT control mouse. The x-axis represents circadian period (hours, 0–40 h), and the y-axis represents Qp value (rhythm strength, a dimensionless unit reflecting the peak amplitude of the periodogram). The red line indicates the significance threshold. Individual periodograms for all animals are provided in the Supplementary Figures S2A, S3B. **(F)** Quantification of rhythm amplitude (Qp value, arbitrary units representing periodogram peak height). **(G)** Average daily total locomotor activity (each data point represents the mean of 7 consecutive days per mouse, counts/day). **(H)** Average light-phase locomotor activity (each data point represents the mean of 7 consecutive light-phases per mouse, counts/day). **(I)** Average dark-phase locomotor activity (each data point represents the mean of 7 consecutive dark-phases per mouse, counts/day). (Paired t-test; *n =* 7 mice measured before and after AAV-TeNT injection. Exact statistics: F, *t* = 4.490, df = 6; G, *t* = 5.659, df = 6; H, *t* = 1.812, df = 6; I, *t* = 6.085, df = 6. **p < 0.01, ***p < 0.001, ns, not significant).

Gross motor activity was measured using an implantable telemetry probe (Starr Life Sciences, Mini-Mitter, G2 E-Mitter). This procedure followed previously established protocols for implantable telemetry-based locomotor activity recording (Zhao et al., 2022). Mice were anesthetized with isoflurane (5% induction, 1–1.5% maintenance), and the abdominal area was shaved and sterilized with 75% ethanol. A small midline incision was made to expose the peritoneal cavity, and the transmitter was inserted into the intraperitoneal space. The abdominal wall and skin were then sutured in layers. Following a postoperative recovery period of at least 7 days, animals were individually housed in cages placed on telemetry receivers (Starr Life Sciences, Mini-Mitter, ER4000 Energizer/Receiver), which powered the transmitters and continuously recorded locomotor activity data at 1-min intervals. Activity “counts” were defined according to the E-Mitter system, in which any change in transmitter signal strength is interpreted as movement, and the total number of such events within each sampling bin (up to 1-s resolution; maximum 10 counts per second) was recorded as a measure of locomotor activity. This measure reflects the frequency of movement events rather than their magnitude or direction. Raw data were imported into ClockLab (v6.1.15) software for analysis, and rhythm amplitude was quantified using Chi-square periodogram analysis. Specifically, rhythm amplitude was defined as the highest peak value obtained from the Chi-square periodogram output in ClockLab. As illustrated in Figure 1A, animals were first recorded for 7 days to establish baseline locomotor activity (Pre period). On day 8, AAV2/9-CMV-DIO-TeNT-2A-GFP was bilaterally injected into the SCN. Following viral injection, locomotor activity recording continued, and days 15–21 were defined as the post-injection period (Post period). Behavioral analyses were performed on *n =* 7 mice per group.

### 2.6 Statistics

All data are presented as mean ± SEM and were analyzed using GraphPad Prism 10. Sample sizes were determined based on previous studies employing similar experimental designs, rather than formal power calculations. Specifically, group sizes were chosen based on previously published studies investigating SCN circuit manipulations and circadian locomotor activity using viral approaches (Xie et al., 2023). Data distributions were assessed for normality using the Shapiro–Wilk test, and homogeneity of variance was assessed using Levene’s test prior to statistical comparisons. All statistical analyses in this study were performed using paired t-tests. Injection accuracy and viral expression were verified in all animals. All animals included in the final analysis were confirmed to have correct viral targeting based on histological verification. No animals were excluded due to mistargeted injections. Sample sizes for each experiment are provided in the corresponding figure legends.

## 3 Results

### 3.1 Inhibition of SCNCCK neurons significantly attenuates the spontaneous locomotor rhythm

To assess whether synaptic outputs from SCN^CCK^ neurons contribute to the regulation of spontaneous locomotor rhythms, we selectively blocked neurotransmission from these neurons by injecting a Cre-dependent tetanus toxin virus into the SCN of CCK-IRES-Cre mice. Locomotor activity was continuously recorded for one week prior to viral injection to establish baseline rhythms, and subsequently monitored for an additional two weeks following surgery (Figures 1A–C). Viral expression was largely restricted to the SCN with minimal spread outside this region (Supplementary Figure S1A).

Before viral manipulation, mice displayed robust daily oscillations in locomotor activity under a 12:12 h LD cycle, as quantified by Chi-square periodogram analysis, with rhythm strength defined as the Qp value (peak height of the periodogram) (Figures 1D,E). Following TeNT-mediated synaptic blockade in CCK-Cre mice, double-plotted actograms revealed a pronounced disruption of daily rhythmic organization, with activity distributed more uniformly across the 24 h cycle, accompanied by an approximately 36% reduction in rhythm strength (Qp value) relative to baseline levels. In contrast, WT mice showed no detectable behavioral phenotype (Figures 1D,F). Consistent with these observations, average daily total locomotor activity in CCK-TeNT mice was significantly reduced by approximately 37% relative to baseline levels. This reduction was primarily driven by an approximately 45% decrease in dark-phase locomotor activity, whereas light-phase locomotor activity remained unchanged. No significant changes were observed in WT control mice (Figures 1G–I). Together, these results indicate that synaptic output from SCN^CCK^ neurons is required for the normal expression of spontaneous locomotor activity rhythms.

### 3.2 SCN^CCK^ neurons project broadly to hypothalamic, thalamic and limbic structure

**Figure 2:**
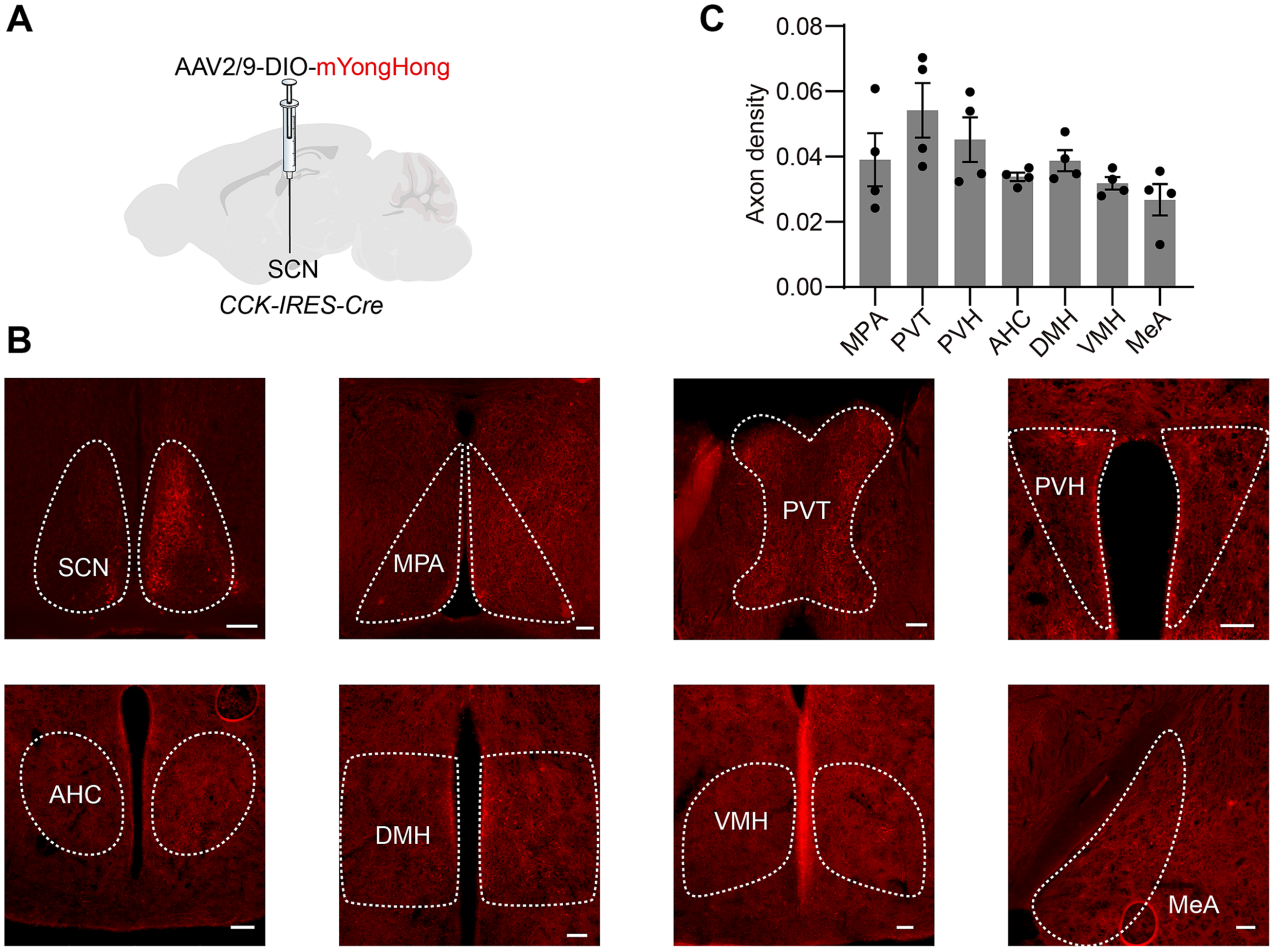
CCK neurons innervate multiple downstream brain regions. **(A)** Schematic illustration of Cre-dependent viral labeling in the SCN of CCK-IRES-Cre mice. **(B)** Representative fluorescence images of unilateral AAV2/9-CAG-DIO-mYongHong injection into the SCN (right hemisphere specified in histological validation; injection side indicated in the corresponding images) and axonal projections of CCK neurons (red) in downstream target regions of the SCN, including medial preoptic area (MPA), paraventricular thalamic nucleus (PVT), paraventricular hypothalamic nucleus (PVH), anterior hypothalamic area (AHC), dorsomedial hypothalamic nucleus (DMH), ventromedial hypothalamic nucleus (VMH), and medial amygdala nucleus (MeA). Scale bars, 100 μm. **(C)** Quantification of axonal density in each target region. Data are presented as mean ± SEM; *n =* 4 mice. Axonal density was quantified in the ipsilateral hemisphere relative to the injection site, and the contralateral hemisphere was used as an anatomical reference where applicable.

To identify potential downstream targets of SCN^CCK^ neurons regulate spontaneous locomotor activity rhythms, we selectively labeled CCK neurons in the SCN by injecting a Cre-dependent mYongHong reporter virus into the SCN of CCK-IRES-Cre mice (Figure 2A). mYongHong is a highly photostable monomeric red fluorescent protein, enabling reliable visualization of long-range axonal projections (Xiong et al., 2025). Importantly, mYongHong expression was specifically localized to the SCN, with no detectable signal observed in adjacent hypothalamic regions, indicating spatially restricted viral expression (Supplementary Figure S1B). Axon-positive fluorescent signals were identified and quantified using QuPath software (Supplementary Figure S4). Whole-brain sectioning combined with fluorescence imaging revealed that SCN^CCK^ neurons project along the anterior–posterior axis to multiple downstream regions, including the medial preoptic area (MPA) (0.039 ± 0.008), paraventricular thalamic nucleus (PVT) (0.054 ± 0.008), paraventricular hypothalamic nucleus (PVH) (0.045 ± 0.007), anterior hypothalamic area (AHC) (0.034 ± 0.001), dorsomedial hypothalamic nucleus (DMH) (0.039 ± 0.003), ventromedial hypothalamic nucleus (VMH) (0.032 ± 0.002), and medial amygdala nucleus (MeA) (0.027 ± 0.005) (Figure 2B). Furthermore, we quantified axonal density in each target region to provide a systematic and quantitative assessment of the whole-brain distribution of SCN^CCK^ neuronal outputs. Projection density varied across downstream regions, with the highest values observed in the PVT and lower values in the VMH and MeA (Figure 2C). This analysis reveals a widespread projection pattern of SCN^CCK^ neurons across hypothalamic, thalamic, and limbic structures, establishing an anatomical framework for downstream circuit mechanisms underlying locomotor rhythm regulation.

## 4 Discussion

In the present study, we demonstrate that synaptic output from SCN^CCK^ neurons is required for the normal expression of spontaneous locomotor rhythms. Selective TeNT-mediated blockade significantly attenuated daily rhythmic organization under LD conditions. Anatomical tracing further revealed that SCN^CCK^ neurons project broadly to multiple hypothalamic, thalamic, and limbic regions, including the MPA, PVT, PVH, AHC, DMH, VMH, and MeA, with varying projection densities across target regions. Together, these findings suggest that SCN^CCK^ neurons play a critical role in regulating locomotor activity rhythms.

Although rhythmic strength was significantly reduced, locomotor rhythms did not disappear entirely. This dissociation likely reflects the hierarchical and redundant organization of SCN output pathways. Early SCN transplantation studies showed that grafted SCN tissue lacking outward synaptic connections could still restore circadian rhythms in SCN-lesioned animals (Ralph et al., 1990), supporting the view that diffusible humoral factors can contribute to SCN output signaling. Our results indicate that locomotor activity rhythmicity is not completely abolished, suggesting that the SCN is still able to convey rhythmic signals to downstream brain regions through diffusible signaling and paracrine mechanisms, thereby providing temporal information via non-synaptic pathways.

At the molecular level, this interpretation is further supported by the mechanism of TeNT, which blocks synaptic transmission by cleaving synaptobrevin/VAMP2, thereby inhibiting vesicle-mediated neurotransmitter release without affecting neuronal survival. This suggests that SCN^CCK^ neurons regulate locomotor rhythmicity through their synaptic outputs within the SCN network, likely involving downstream neuromodulatory and endocrine signaling pathways that collectively convey circadian information, although the specific molecular mediators remain to be identified in future studies.

Our axonal tracing revealed broad projections to several hypothalamic, thalamic, and limbic nuclei; however, conventional section-based imaging may underestimate the full extent of SCN^CCK^ connectivity. Tissue clearing and whole-brain light-sheet imaging approaches have demonstrated far more extensive long-range projections from genetically defined neuronal populations than previously appreciated. Applying brain-wide clearing methods such as iDISCO or CLARITY could uncover additional sparse or collateral projections that are difficult to detect using traditional fluorescence microscopy (Chung et al., 2013; Renier et al., 2014). Such approaches would allow more comprehensive quantification of projection density and potentially reveal target-specific biases relevant to behavioral regulation.

While our current results highlight an effect of SCN^CCK^ neuron inhibition on locomotor activity, the circadian system orchestrates multiple other physiological processes, including sleep–wake cycles, body temperature, feeding, and drinking rhythms (Starnes and Jones, 2023). These outputs often follow daily rhythms coordinated by SCN signals but may involve distinct downstream pathways. Future studies incorporating additional behavioral paradigms, including food intake, water consumption, motor coordination, affective behavioral assessments, and direct measurements of sleep–wake architecture, may help determine whether the reduced locomotor activity observed following SCN^CCK^ neuronal silencing reflects altered circadian output alone or additional changes in motor, emotional, or sleep-related processes. Such comprehensive phenotyping will help clarify whether CCK neurons selectively regulate specific physiological rhythms.

Beyond locomotor activity, our findings may also have implications for understanding the neural mechanisms linking circadian timing to sleep–wake regulation. The SCN serves as the master circadian pacemaker coordinating behavioral and physiological rhythms, and disruption of specific SCN neuronal subpopulations may alter downstream neural states that influence sleep–wake organization. Although sleep was not directly examined in the present study, the identification of SCN^CCK^ neurons as contributors to circadian locomotor rhythmicity provides a foundation for future investigations into how genetically defined SCN circuits participate in adaptive sleep–wake dynamics and broader circadian regulation.

A limitation of the present study is that both male and female mice were included without systematic balancing across experimental groups, and sex was not analyzed as a biological variable. Therefore, potential sex-dependent effects on SCN^CCK^-mediated regulation of locomotor rhythms cannot be excluded. In addition, we did not directly validate the efficacy of TeNT-mediated synaptic blockade using electrophysiological recordings or independent functional assays, and the extent of functional synaptic silencing therefore remains to be confirmed in future studies. Finally, cell-type specificity of Cre-dependent viral expression was inferred based on the CCK-IRES-Cre driver line without direct molecular validation using CCK immunohistochemistry. Although spatial restriction of viral expression within the SCN was confirmed histologically, potential low-level Cre-line leakage or off-target recombination, as well as Cre-independent viral expression in the absence of WT controls, cannot be fully excluded. Future studies incorporating electrophysiological validation, molecular co-labeling, and appropriate control injections will further strengthen the mechanistic and anatomical specificity of these findings.

## 5 Conclusion

In summary, our findings demonstrate that synaptic output from SCN^CCK^ neurons is required for the normal expression of spontaneous locomotor activity rhythms. Disruption of these neurons weakens rhythmic strength and selectively reduces dark-phase activity without abolishing overall rhythmicity, indicating partial compensation via non-synaptic SCN outputs. Anatomically, SCN^CCK^ neurons exhibit broad projections across hypothalamic, thalamic, and limbic regions. Together, these results identify SCN^CCK^ neurons as an important component of SCN output pathways contributing to the robustness of circadian locomotor rhythms.

## Data Availability

The original contributions presented in the study are included in the article/Supplementary material, further inquiries can be directed to the corresponding author.
