# Supplementary material for: Synaptic output from suprachiasmatic nucleus cholecystokinin neurons regulates locomotor rhythmicity
Source: Front Neurosci. 2026 Jul 3;20:1882096. doi: 10.3389/fnins.2026.1882096 (PMC13375715; doi:10.3389/fnins.2026.1882096)
Supplement: Supplementary file 1 [file Data_sheet_1.docx]

Supplementary Material

## Supplementary Figures


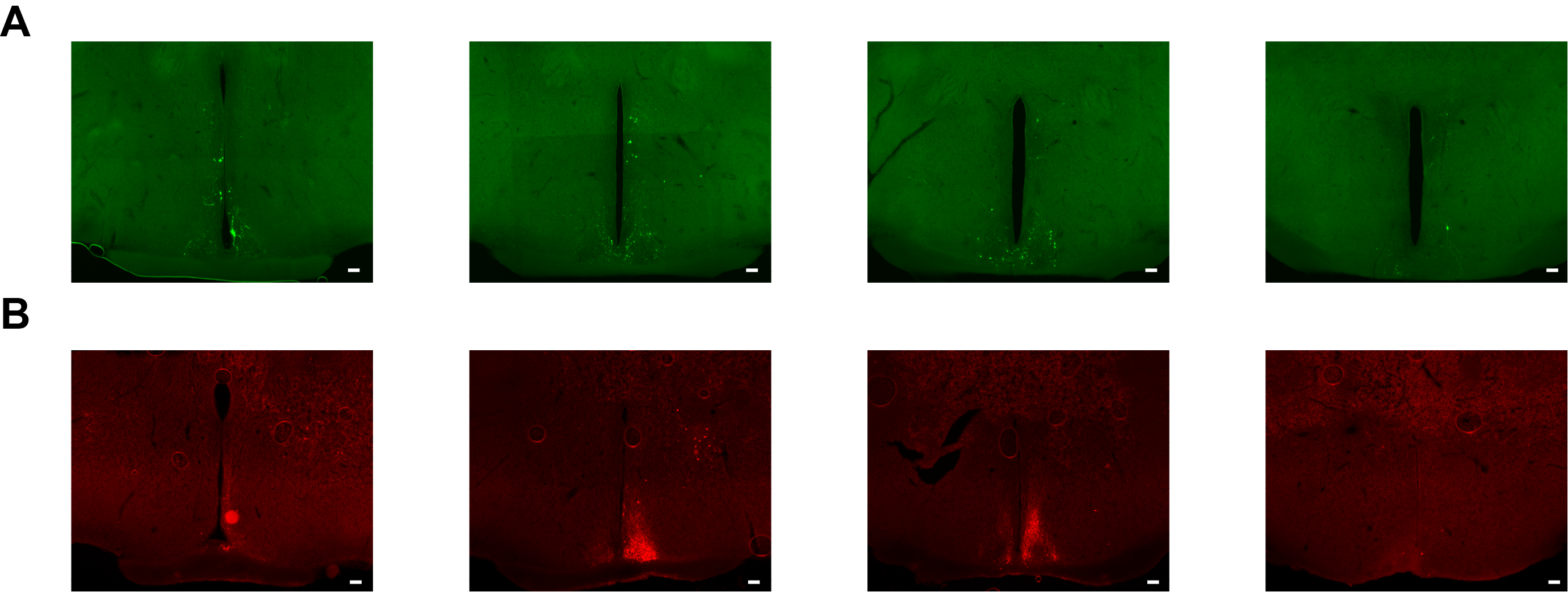


**Supplementary Figure 1.** **Verification of SCN-targeted viral expression**

**(A)** Representative coronal hypothalamic sections from anterior to posterior SCN showing bilateral AAV2/9-CMV-DIO-TeNT-GFP expression in the SCN of CCK-IRES-Cre mice. GFP expression was restricted to the SCN injection site. Scale bar, 100 μm. **(B)** Representative coronal hypothalamic sections from anterior to posterior SCN showing unilateral AAV2/9-CAG-DIO-mYongHong expression in the SCN of CCK-IRES-Cre mice. mYongHong fluorescence was confined to the SCN region. Scale bar, 100 μm.


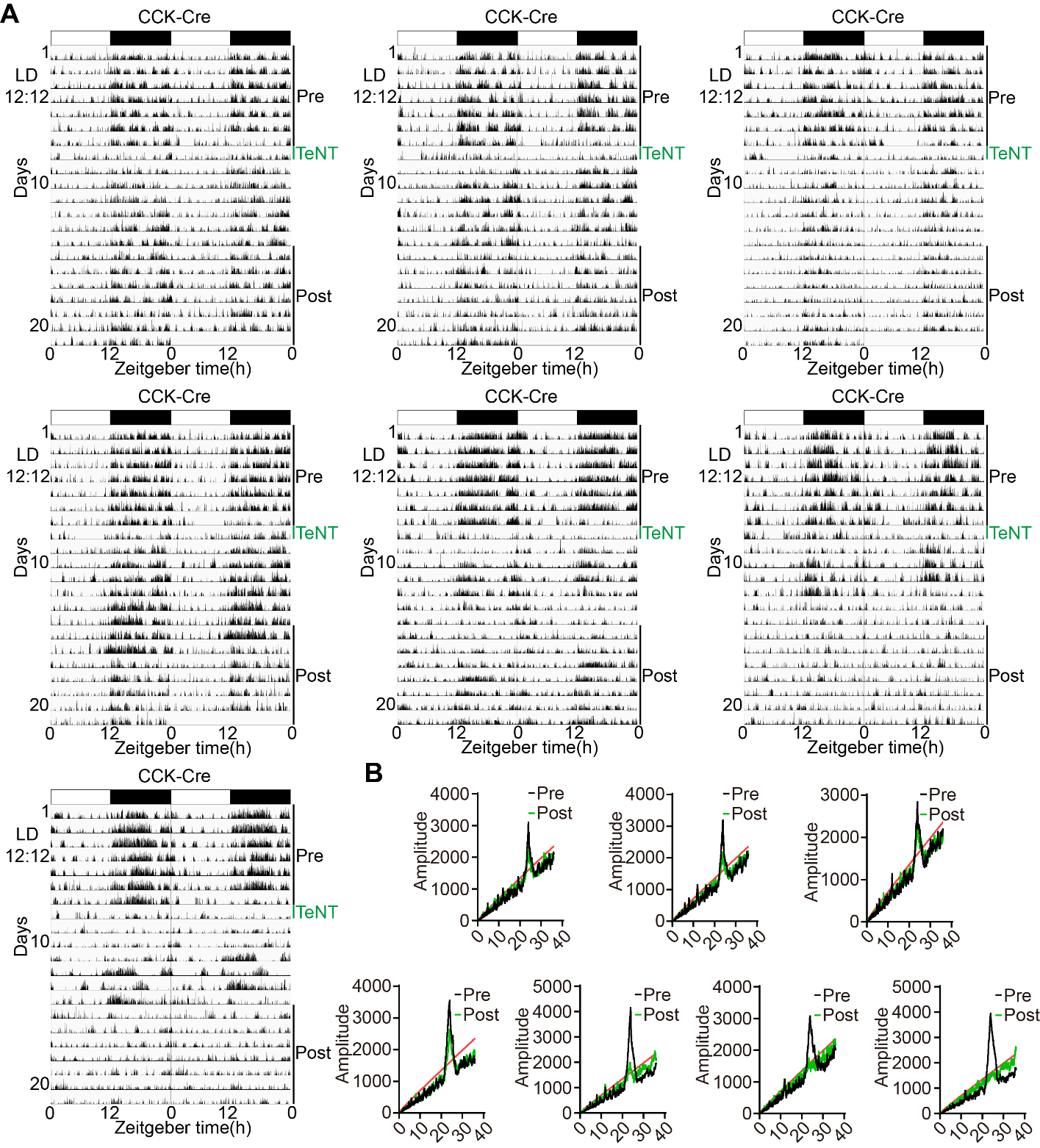


**Supplementary Figure 2.** **Silencing of SCN^CCK^ neurons attenuates circadian locomotor activity rhythms under light–dark conditions.**

**(A)** Double-plotted actograms showing locomotor activity rhythms of individual SCN CCK-TeNT mice maintained under 12:12 h light–dark (LD) conditions. **(B)** Corresponding Chi-square periodogram analyses of the double-plotted actograms shown in panel A.


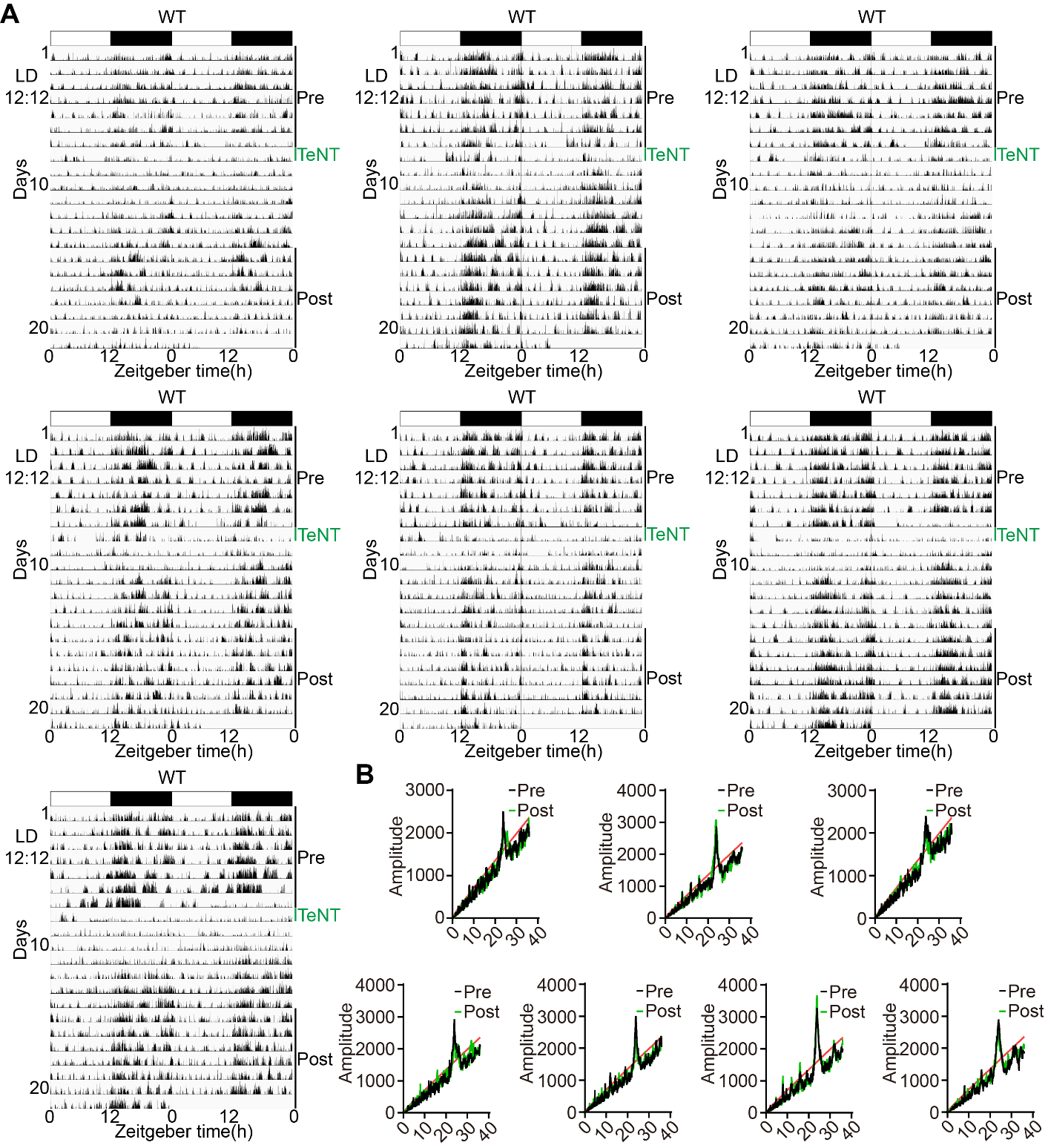


**Supplementary Figure 3.** **WT mice retain normal circadian locomotor rhythms.**

**(A)** Double-plotted actograms showing locomotor activity rhythms of individual WT-TeNT mice maintained under 12:12 h light–dark (LD) conditions. **(B)** Corresponding Chi-square periodogram analyses of the double-plotted actograms shown in panel A.

**
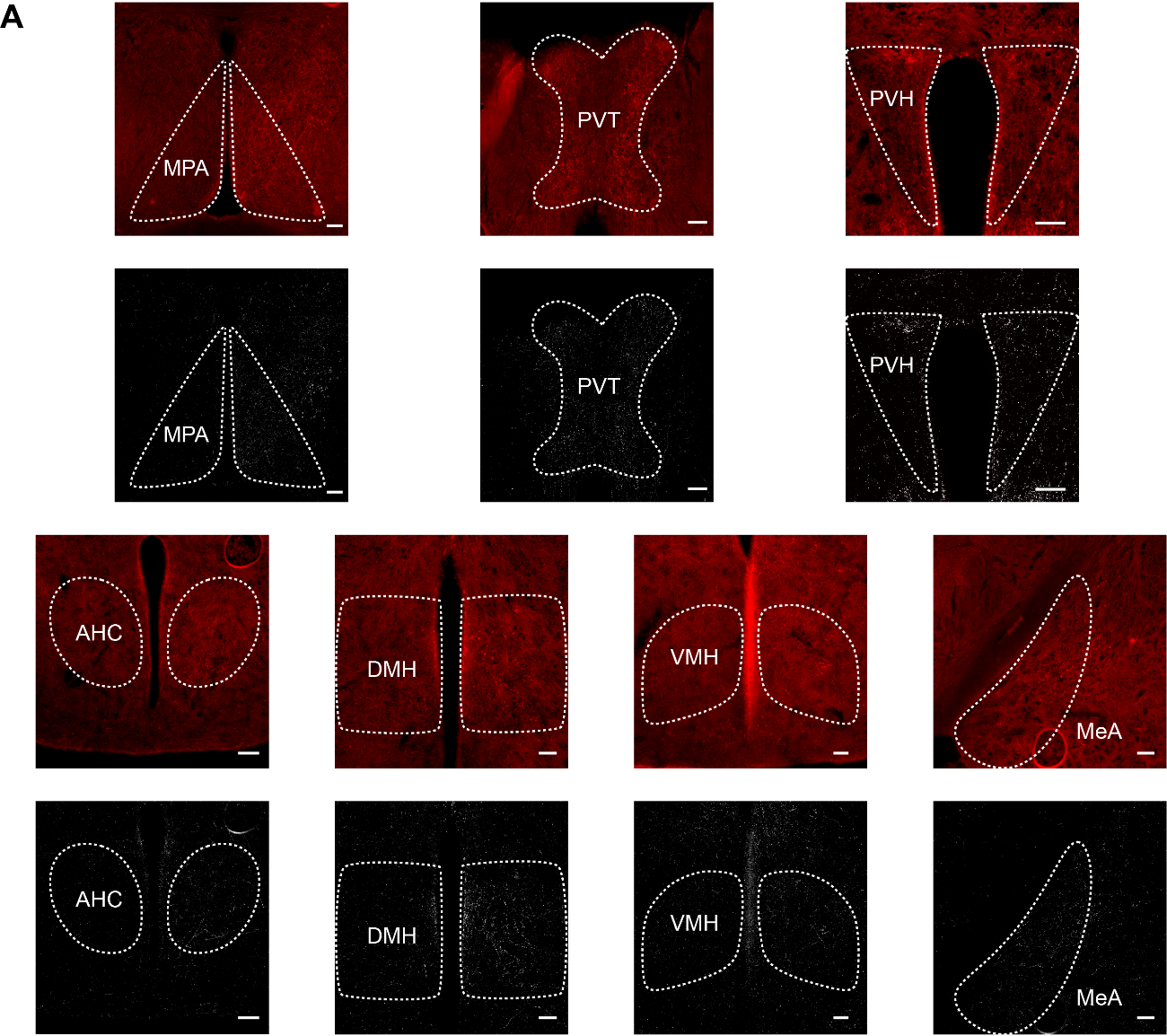
**

**Supplementary Figure 4. Brain-wide downstream projections of SCN^CCK^ neurons and corresponding axonal labeling**

(A) Representative fluorescence images of unilateral AAV2/9-CAG-DIO-mYongHong injection into the SCN and axonal projections of CCK neurons (red) in downstream target regions of the SCN. Binary output of the QuPath pixel classifier used for axon density quantification. Axon-positive pixels identified by the trained classifier are shown in white, and background pixels are shown in black. No raw fluorescence image is displayed. Regions of interest (ROIs) are overlaid for anatomical reference.
